# Prevalence trends of hypertension among 9–17 aged children and adolescents in Yunnan, 2017–2019: a serial cross-sectional surveillance survey

**DOI:** 10.1186/s12889-021-10258-1

**Published:** 2021-02-12

**Authors:** Yunjuan Yang, Jieqing Min, Litao Chang, Jiajia Chai, Zhizhong Song, Shun Zha, Min Zhang, Hong Liu, Fan Yang

**Affiliations:** 1 Department of School Health, Yunnan Provincial Center for Disease Control and Prevention, NO. 158 Dongsi Street, Kunming, 650022 Yunnan Province China; 2 Kunming University of Medical, Kunming, 650500 Yunnan Province China; 3 Kunming Children’s Hospital, Kunming, 650228 Yunnan Province China; 4 International Cooperation Office, City College, Kunming University of Science and Technology, Kunming, 650051 Yunnan Province China; 5 Department of Public Health, Pu’er Center for Disease Control and Prevention, NO.11 Chayuan Road, Pu’er, 665000 Yunnan Province China

**Keywords:** Hypertension, Children, Adolescent, Epidemic trend, Prevalence, Cross sectional survey

## Abstract

**Background:**

We aim to describe the prevalence and trends of hypertension among 9–17 school-aged students from 2017 to 2019 in Yunnan, China.

**Methods:**

We conducted a cross-sectional study by using data from the Students’ Health Surveillance Surveys of 2017, 2018 and 2019 in Yunnan. The Students’ Health Surveillance Surveys in Yunnan collected date on physical fitness and health status of students in Yunnan through multistage-stratified sampling in 3 prefectures. In each prefecture, the study population were classified by gender and region (urban or rural), and each group had an equal size. Diagnosing criteria of hypertension was set with reference to Chinese age-specific and height-specific blood pressure (BP), to identify the abnormal status of boys and girls separately. ANOVA test was adopted to measure the differences in the mean BP stratified by gender, age, prefecture and area, and Chi-square test was used to compare the percentages of hypertension in different areas. For comparability, the age-standard and gender-standard population prevalence was calculated by directly using China Census in 2010 as a standard population. Totally 24,890 participants aged 9–17 years were included in this study.

**Results:**

From 2017 to 2019, there were 24,872 students completed physical examinations and included in the analysis, of which 3288 were diagnosed with hypertension. The total prevalence of hypertension was 13.72, 12.49 and 13.45% among 9–17 years school-aged population in Yunnan, respectively. The total age-standardized hypertension prevalence trended to decrease from 13.82 to 13.48%. For urban population, the age-standardized hypertension prevalence decreased slightly from 11.24 to 10.13%. While, for rural population, it increased from 17.58 to 19.16%. The average annual growth rate in rural population was 0.53%.

**Conclusions:**

From 2017to 2019, there was a significant and continuous increase in the prevalence of hypertension in 9–17 years school-aged population in Yunnan. Hypertension is epidemic among children and adolescents in Yunnan. We should take effective and comprehensive intervention measures to reduce its prevalence among school-aged children.

## Background

With the rapid development of social economy and changing of people’s lifestyle, hypertension has become the most common chronic non-communicable disease globally. According to a WHO report, of the estimated 1.13 billion people with hypertension, less than 1 in 5 has it under control [1]. Increased blood pressure is a leading risk for death and disability globally. Hypertension is also a major cause of premature death worldwide, with upwards of 1 in 4 men and 1 in 5 women, over a billion people, having the condition [1–4].

In recent years, hypertension has shown a trend of prevalence at younger age [5]. Hypertension was once rare in children and adolescents. But it has raised a serious public health challenge to us [6, 7]. The prevalence of hypertension in Chinese children and adolescents has increased from 7.1% in 1991 to 13.8% in 2009, with an average annual rate of 0.47% [8, 9]. And more and more studies have found that the origin of hypertension in adulthood could be traced back to hypertension in childhood [7, 10–13]. Children with elevated blood pressure were more likely to become hypertensive adults [14, 15]. Hypertension in children and adolescents is a serious medical condition, and a risk factor of atherosclerotic disease, stroke, diabetes, kidney failure and blindness [16, 17]. Therefore, it is important to identify childhood hypertension timely. And its prevention become critical to control and curb the rising trend of hypertension, cardiovascular and cerebrovascular diseases in adults.

This study aimed to estimate the prevalence of hypertension in children and adolescents in Yunnan, located in Southeast of China, and understand its trend in recent 3 years. We explored different characteristics associated with hypertension to inform effective strategies and measures in secondary prevention of hypertension in children and adolescents, to promote primary prevention of serious cardiovascular events such as hypertension and coronary heart diseases in adulthood. This study used data from the Yunnan Students’ Health Surveillance Surveys conducted from 2017 to 2019.

## Methods

### Study design and data source

**Fig. 1:**
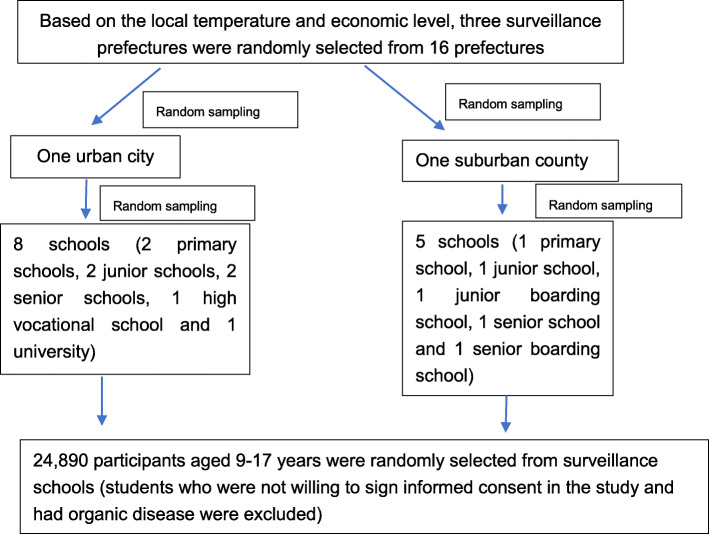
Sampling flowchart

The study was a cross-sectional study with data that were derived from the Yunnan Students’ Health Surveillance Surveys from 2017 to 2019 [18]. In brief, the study participants were randomly sampled from three surveillance prefectures in Yunnan (namely, Kunming, Honghe and Pu’er). Kunming is the capital of Yunnan, Honghe is in the South of Yunnan, and Pu’er is in the South-west of Yunnan. The average elevation of three surveillance prefectures was 1809 m in Kunming, 1453 m in Honghe and 1303 m in Pu’er. And the annual average temperature of the three prefectures was 12 ~ 25 °C in Kunming, 15 ~ 25 °C in Honghe and 14 ~ 26 °C in Pu’er, respectively. One urban city and one suburban county were randomly selected from each prefecture. In the urban city, we randomly selected eight schools as the surveillance schools, and in suburban county, five schools were randomly selected as the surveillance schools (include one junior boarding school and one senior boarding school). Surveillance schools remained consistent from 2017 to 2019. The participants who have diseases in the heart, liver, kidney, and other major organs were exclude. Participants who were not willing to sign informed consent were also exclude. Other all participants aged 9–17 years were randomly selected from surveillance schools (as in Fig. 1). And all participants and/or their parents/guardians provided written informed consent. There were 24,890 participants aged 9–17 years sampled in this study, account of it, in 2017 (*n* = 7828), 2018 (*n* = 8165) and 2019 (*n* = 8897). In total, there were 12,096 boys and 12,794 girls included.

### Measurements and definitions

The survey included a standardized physical examination, which were conducted by well-trained health workers who followed a reference protocol recommended by China Center for Disease Control and Prevention and built up a mobile examination team.

Physical examinations included measurement and record of individuals’ height and weight. Height was measured to the minimal unit of 0.1 cm by a metal column Height and Sitting Height Measuring Instrument while the students did not wear shoes (Jianmin Height and Sitting Height Meter JH-1200). Weight was measured to the minimal unit of 0.10 kg by a balance-beam scale while the students wore light-weighted clothing (Jianmin Weighing Scale RCS-160II). Both the scales and stadiometers were calibrated before use [6, 15, 18, 19]. Body mass index (BMI) was calculated as weight in kilograms divide by height in meters squared. Blood pressure (BP) was measured in accordance with the recommendation of the National High Blood Pressure Education Program (NHBPEP) Working Group in Children and Adolescents [20], by using an auscultation mercury sphygmomanometer by the trained and qualified personnel who followed a standard protocol as described elsewhere [21]. BP was measured after resting at least 5 min in the sitting position. An appropriately sized cut off was used to measure the BP of each child on their right arm (Oumulong HEM-7211Electronic Sphygmomanometer and Mindray Children’s Blood Pressure Cuff CM1202). Systolic Blood Pressure (SBP) was determined by onset of the first ‘tapping’ Korotkoff sound (K1), and Diastolic Blood Pressure (DBP) was determined by the fifth Korotkoff sound (K5). The mean of three measurements was used for analysis. Height is an identified important factor in the process of establishing pediatric HBP references worldwide [22, 23]. Therefore, this study used the Chinese age-specific and height-specific BP references to detect the abnormal status of boys and girls separately [24]. HBP was defined as SBP and/or DBP at least 95th percentile based on age, sex, and height percentiles [20, 24].

### Statistical analyses

The primary data analysis was conducted in 2020. Differences in the mean BP by age, gender and area were compared using the ANOVA test. The prevalence estimates for hypertension in different survey years (2017, 2018 and 2019) were calculated by age, gender, prefecture, and area (urban and rural) subgroups. Division of urban and rural was based on the Chinese Administrative Division. Univariate methods were not used to estimate *P*-value for differences by gender, age group, and area group, because high statistical power was achieved from the large sample sizes.

For comparability, the age-standardized and gender-standardized prevalence was calculated by directly using the Chinese Census 2010 as a standard population. The age groups were set as 9–11 years old, 12–14 years old and 15–17 years old. Chi-square analyses were conducted to assess differences in prevalence of hypertension for categorical variables. All data were analyzed using SPSS 21.0 software. All the analyses included sample weights that accounted for the unequal probabilities of selection, oversampling, and non-response. Statistical significance was defined as*P* < < 0.05.

## Results

### Population demography

As shown in Table 1, the study sample was recruited from primary, junior and senior high schools in 3 prefectures of Yunnan, China. A total of 24,890 children and adolescents of 9–17 years old were included from 2017 to 2019. And 24,872 of them completed physical examinations, with the complete rate of 99.93%. All of those who had completed physical examination were included in the analysis, with 12,091 boys and 12,781 girls. The average age was 13.11 ± 2.50 years old. No significant difference was observed in the distribution of participants from both urban and rural areas from 2017 to 2019. These data were comparable. And there were 14,300 urban and 10,572 rural participants. Significant difference was noted in the distribution of participants with different gender, age groups and from different prefectures from 2017 to 2019 (*P* < < 0.05).

**Table 1:** The demographic characteristics of students in the study, 2017–2019

Index	2017	2018	2019	***χ***^***2***^	***P***
**All**	7824	8163	8892		
**Gender**				8.825	0.012
Boy	3697	3989	4405		
girl	4122	4172	4487		
**Age**				28.056	0.000
9–11	2292	2510	2939		
12–14	2828	2904	3068		
15–17	2699	2747	2885		
**Prefecture**				9.932	0.042
Kunming	2529	2680	2963		
Honghe	2763	2784	2937		
Pu’er	2527	2697	2992		
**Area**				0.502	0.778
urban	4519	4690	5091		
rural	3300	3471	3801		

### Secular changes in BP

**Fig. 2:**
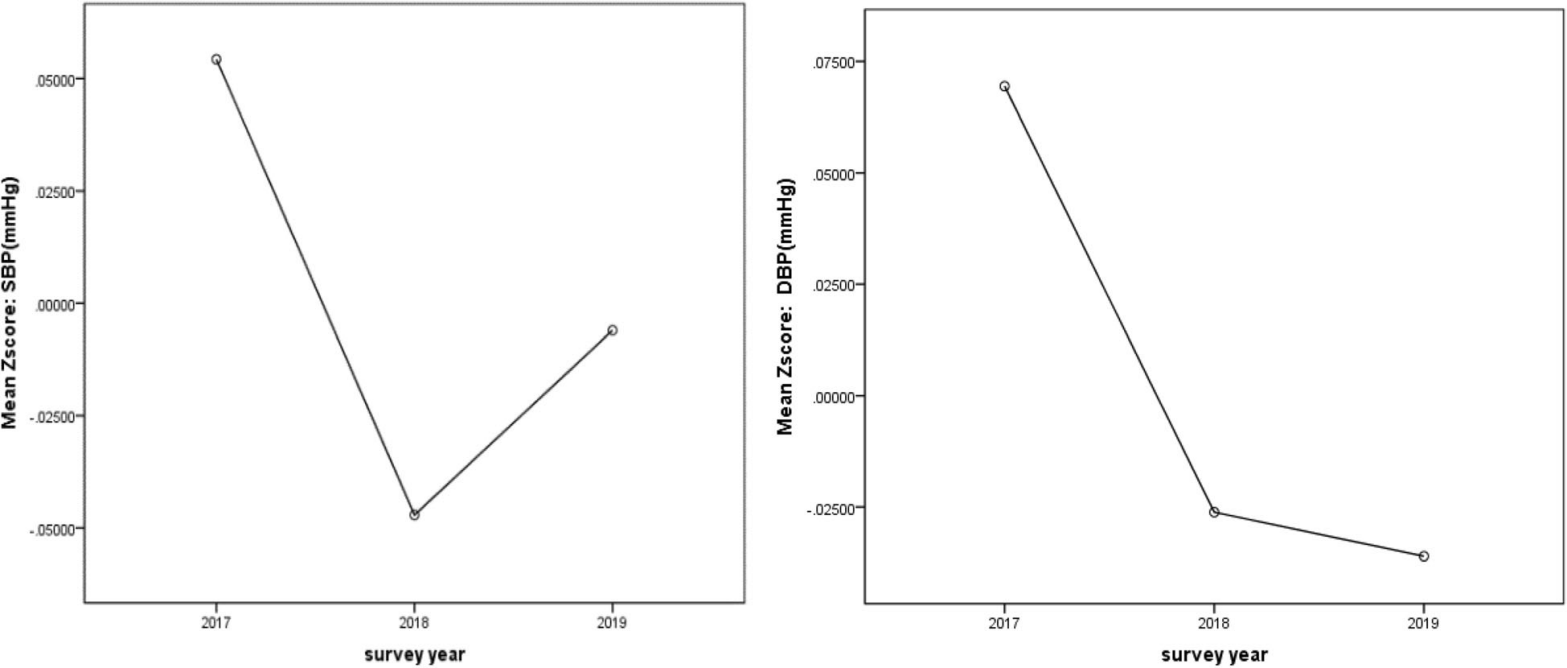
Overall trend of standard blood pressure (SBP and DBP) in aged 9–17 years in Yunnan, 2017–2019.(by using the ANOVA test, *P*<0.01)

As shown in Fig. 2, a greater decrease trend was observed in the overall standardized DBP (*P* < 0.01), compared to the standardized SBP (*P* < 0.01), from 2017 to 2019.

As shown in Table 2, from 2017 to 2019, in urban areas, the mean standardized blood pressure, both SBP and DBP, decreased in all students (*P* < 0.01). However, in rural areas, the mean standardized SBP increased in all age groups and genders (*P* < 0.01), while the mean standardized DBP decreased obviously (*P* < 0.01).

**Table 2:** the blood pressure characteristics of different area, 2017–2019. ** *P* value<0.01. (SBP: Systolic Blood Pressure; DBP: Diastolic Blood Pressure)

Subgroup	***SBP***			***F***	***DBP***			***F***	***Zscore:SBP***			***F***	***Zscore:DBP***			***F***
	2017	2018	2019		2017	2018	2019		2017	2018	2019		2017	2018	2019	
**Urban**				43.969**				27.168**				43.969***				27.168**
9-11y	101.65 ± 12.16	102.52 ± 11.98	101.12 ± 12.96		63.11 ± 8.71	63.44 ± 8.68	61.49 ± 8.69		−0.38 ± 0.97	−0.31 ± 0.95	−0.42 ± 1.03		−0.34 ± 0.92	−0.30 ± 0.92	− 0.51 ± 0.92	
12-14y	107.70 ± 10.81	108.98 ± 12.11	106.59 ± 11.74		64.59 ± 8.26	64.09 ± 9.17	64.25 ± 8.04		0.10 ± 0.86	0.20 ± 0.96	0.01 ± 0.93		−0.18 ± 0.88	− 0.23 ± 0.97	− 0.22 ± 0.85	
15-17y	113.18 ± 11.54	111.04 ± 11.94	109.43 ± 11.41		68.16 ± 8.36	66.52 ± 8.85	66.44 ± 8.32		0.54 ± 0.92	0.37 ± 0.95	0.24 ± 0.91		0.20 ± 0.89	0.02 ± 0.94	0.02 ± 0.88	
boys	110.75 ± 13.10	110.15 ± 13.04	108.37 ± 13.14	404.578**	65.40 ± 8.86	64.21 ± 9.33	63.82 ± 8.82	49.224**	0.34 ± 1.04	0.30 ± 1.04	0.15 ± 1.04	404.578**	−0.09 ± 0.94	−0.22 ± 0.99	− 0.26 ± 0.93	49.224**
girls	106.30 ± 11.30	106.21 ± 11.66	104.35 ± 11.43		65.98 ± 8.53	65.64 ± 8.64	64.89 ± 8.32		−0.01 ± 0.90	− 0.02 ± 0.93	− 0.17 ± 0.91		− 0.03 ± 0.90	− 0.07 ± 0.92	− 0.15 ± 0.88	
Total	108.42 ± 12.39	108.13 ± 12.50	106.25 ± 12.43		65.71 ± 8.69	64.09 ± 9.01	64.39 ± 8.58		0.16 ± 0.99	0.14 ± 0.99	−0.02 ± 0.99		−0.06 ± 0.92	−0.14 ± 0.95	− 0.20 ± 0.91	
**Rural**				770.170**				425.911*				770.170**				425.911*
9-11y	100.03 ± 11.44	96.30 ± 10.64	101.60 ± 11.11		65.76 ± 9.87	63.44 ± 9.54	65.35 ± 9.44		−0.51 ± 0.91	−0.81 ± 0.85	− 0.38 ± 0.88		−0.06 ± 1.05	− 0.30 ± 1.01	− 0.10 ± 1.00	
12-14y	106.34 ± 11.90	104.93 ± 11.84	108.74 ± 11.45		69.15 ± 9.91	68.77 ± 9.16	68.57 ± 9.58		−0.01 ± 0.95	−0.12 ± 0.94	0.18 ± 0.91		0.30 ± 1.05	0.26 ± 0.97	0.24 ± 1.02	
15-17y	110.97 ± 11.47	109.31 ± 12.93	111.90 ± 11.50		71.77 ± 9.86	71.90 ± 9.88	72.29 ± 9.28		0.36 ± 0.91	0.23 ± 1.03	0.43 ± 0.91		0.58 ± 1.04	0.59 ± 1.05	0.64 ± 0.98	
boys	106.83 ± 13.01	104.21 ± 13.08	107.64 ± 12.36	127.403**	69.17 ± 10.60	68.16 ± 10.27	67.74 ± 9.92	5.822**	0.03 ± 1.03	−0.18 ± 1.04	0.10 ± 0.98	127.403**	0.31 ± 1.12	0.20 ± 1.09	0.15 ± 1.05	5.822**
girls	104.03 ± 11.62	101.36 ± 12.28	105.26 ± 11.63		68.18 ± 9.70	66.92 ± 9.74	68.38 ± 9.68		−0.19 ± 0.92	−0.40 ± 0.98	− 0.09 ± 0.92		0.20 ± 1.03	0.07 ± 1.03	0.22 ± 1.03	
Total	105.34 ± 12.37	102.76 ± 12.76	106.52 ± 12.08		68.64 ± 10.14	67.53 ± 10.02	68.04 ± 9.81		−0.09 ± 0.98	−0.29 ± 1.01	0.01 ± 0.96		0.25 ± 1.07	0.13 ± 1.06	0.19 ± 1.04	

From 2017 to 2019, the mean SBP in urban participants decreased from 108.42 mmHg to 106.25 mmHg, while it increased from 105.34 mmHg to 106.52 mmHg in rural population. In terms of the mean DBP, similar pattern of decrease was observed among urban students (from 65.71 mmHg to 64.39 mmHg), while in rural areas, it slightly decreased from 68.64 mmHg to 68.04 mmHg. We also observed that the mean SBP for boys in the urban was higher than that in the rural. However, the mean DBP for boys in the urban was lower than that in the rural. And the mean SBP/DBP for girls in the rural was higher than that in the urban areas (*P* < 0.05).

### The prevalence of hypertension

The crude prevalence of hypertension in total participants was 13.22% (3288/24,872) among students aged 9–17 years in Yunnan. And the standardized prevalence was 13.13%.

From 2017 to 2019, the crude prevalence of hypertension among children and adolescents aged 9–17 years were 13.72% (1073/7819), 12.49% (1019/8161) and 13.45% (1196/8892), respectively in Yunnan (*χ*^*2*^ = 5.966, *P* = 0.051). Prevalence difference was noted in different years. After standardization, the prevalence was adjusted as 13.82, 12.39 and 13.48%, respectively. The standardized prevalence was slightly lower than the crude one. The average annual decrease rate was 0.11%. The average standardized prevalence of hypertension was 13. 23% among students aged 9–17 years over 3 years.

As shown in Table 3, in different age groups, the crude prevalence of children among 12–14 years increased from 13.22 to 14.11% in Yunnan. However, crude prevalence decreased in other age groups.

**Table 3:** Trends in prevalence of hypertension among 9–17 aged children and adolescents in Yunnan, 2017–2019

Index	2017			2018			2019			AAI(%)	***χ***^***2***^	***P***
	N	hypertension	%	N	hypertension	%	N	hypertension	%			
**All**	7819	1073	13.72	8161	1019	12.49	8892	1196	13.45	−0.09		
**Gender**											1.053	0.305
Boy	3697	482	13.04	3989	445	11.16	4405	644	14.62	0.53		
Girl	4122	591	14.34	4172	574	13.76	4487	552	12.30	−0.68		
**Age**											29.173	0.000
9–11	2292	273	11.91	2510	257	10.24	2939	361	12.28	0.12		
12–14	2828	374	13.22	2904	408	14.05	3068	433	14.11	0.29		
15–17	2699	426	15.78	2747	354	9.45	2885	402	13.93	−0.62		
**Prefecture**											380.017	0.000
Kunming	2529	178	7.04	2680	204	7.61	2963	289	9.75	0.90		
Honghe	2763	439	15.89	2784	424	15.23	2937	233	7.93	−2.65		
Pu’er	2527	456	18.05	2697	391	14.50	2992	674	22.53	1.49		
**Area**											150.001	0.000
urban	4519	513	11.35	4690	537	11.45	5091	517	10.16	−0.40		
rural	3300	560	16.97	3471	482	13.89	3801	679	17.86	0.30		

The crude prevalence of hypertension in boys was 13.04% (482/3697), 11.16% (445/3989) and 14.62% (644/4405), respectively (*χ*^*2*^ = 22.229, *P* = 0.000). While, the crude prevalence of hypertension in girls was 14.34% (591/4122), 13.76% (574/4171) and 12.30% (552/4487), respectively (*χ*^*2*^ = 8.215, *P* = 0.016). We observed a significant difference of prevalence in different year either in boys or girls. But there was no statistically significant difference between the prevalence in boys and in girls. The standardized prevalence in boys was 13.03% (2017), 11.23% (2018) and 14.89% (2019) respectively. And that in girls was 14.48% (2017), 13.54% (2018) and 12.24% (2019) respectively. The boys’ hypertension prevalence trended to increase with the average annual increase rate of 0.62%.

In different areas, the crude prevalence of hypertension in the 9–17 years old urban children and adolescents were 11.35% (513/4519), 11.45% (537/4690) and 10.16% (517/5091) respectively (*χ*^*2*^ = 5.245, *P* = 0.073). There was no statistically significant difference in the prevalence of urban population in different years. Whereas the rural crude prevalence of hypertension was 16.97% (560/3300), 13.86% (482/3471) and 17.86% (679/3801) respectively (*χ*^*2*^ = 22.737, *P* = 0.000). A significant difference was observed in the prevalence of rural population in different years. The standardized prevalence of urban students was 11.24% (2017), 11.56% (2018) and 10.13% (2019) respectively. And the rural one was 17.58% (2017), 14.36% (2018) and 19.16% (2019) respectively. And in 3 years, the standardized prevalence in urban was 10.91%, that of rural was 17.12% (*χ*^*2*^ = 150.001, *P* = 0.000). The hypertension prevalence in rural increased over the past 3 years.

In different prefectures, the average crude prevalence of hypertension in 9–17 years children and adolescents were 18.36% (Pu’er), 13.02% (Honghe) and 8.13% (Kunming) (*χ*^*2*^ = 380.02, *P* = 0.000). A significant difference was noted in the prevalence of different year in different prefectures. A trend of decreased hypertension prevalence was observed in Honghe, with the average annual decrease rate of 2.65%. However, the prevalence in both Pu’er and Kunming increased. The crude prevalence of hypertension increased from 18.05% (2017) to 22.53% (2019) in Pu’er, with the average annual increase rate of 1.49%. And that increased from 7.04% (2017) to 9.75% (2019) in Kunming, with the average annual increase rate of 0.90%.

**Fig. 3:**
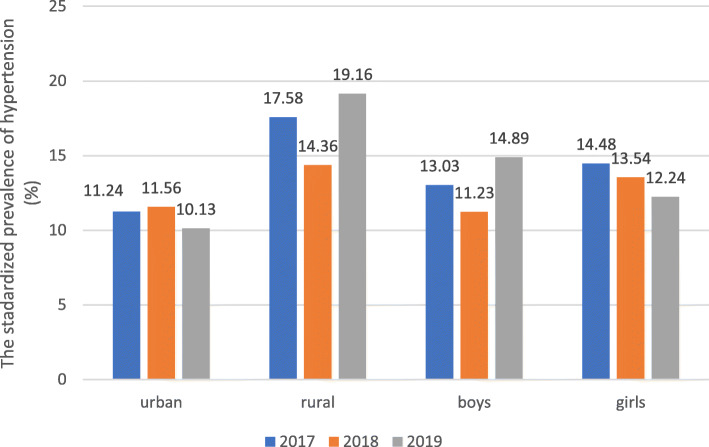
Overall trend of standardized hypertension prevalence in aged 9–17 years in Yunnan, 2017–2019. (by using Chi-square analyses, *P*<0.01)

As shown in Fig. 3, the standardized prevalence of boys’ hypertension increased faster, with the average annual increase rate of 0.62%. And the standardized hypertension prevalence also increased in rural population, with the average annual increase rate of 0.53%.

## Discussion

Our study results indicated that the standardized prevalence of hypertension among children and adolescents aged 9–17 years decreased slightly from 13.82 to 13.48% from 2017 to 2019 in all prefectures, with the average annual decrease rate of 0.11%. The average standardized prevalence of hypertension was 13.23% in the 3 years. This small decrease might be explained by the “white-coat” effects [25, 26], or anxiety that could lead to the increased BP levels. But it might also indicate that hypertension began to affect less children and adolescents in Yunnan. This study result differed from a previous study. That study has reported the prevalence of hypertension in Chinese children and adolescents had increased from 8.9% (1991) to 20.52% (2015) [27]. The study in Yunnan 2014 showed the standardized prevalence of hypertension was 4.26% [28]. Therefore, we should continue to monitor the children and adolescents’ hypertension for a long term. This can help us to know more about its epidemic or trend in Yunnan, to keep a close eye on its actual prevalence trend, to adjust our intervention strategies in time.

Although the total trend of hypertension prevalence decreased, the crude prevalence of children of 12–14 years in Yunnan increased from 13.22 to 14.11%. We should focus on this population and make further research to determine whether transient increases in blood pressure are due to adolescent hormone levels.

In addition, a previous study in Yunnan, the prevalence of hypertension in girls was higher than that of boys. This study results in 2017 and 2018 were consistent with previous study in Yunnan [29]. But it differed from most of the study findings [30–32]. However, the prevalence of hypertension in boys was higher than that of girls for the first time in 2019 in Yunnan, which was consistent with the national study [30]. It may be caused by the heredity, hormones, activity level and dietary and so on. It is necessary to continue paying attention to the changes in hypertension of students in different genders, and to explore the causes of the differences in blood pressure between boys and girls.

The standardized prevalence of hypertension among 9–17 aged children and adolescents in Yunnan (13.48% in 2019) was higher than that of Korean (9.0%), US (1.6%), Brazil (4.5%) and Cameroon (1.6%) levels [32–35]. This study founding is a warning that the hypertension epidemic remains at a relatively high level, compared with children and adolescents in other countries. We shall attach great attention in dealing with this problem. Previous studies confirmed that childhood hypertension not only affected children’s health, but also had a “trajectory” of blood pressure. In other words, childhood hypertension can gradually develop into adult hypertension and increase the risk of cardiovascular disease in adulthood [7, 10, 15, 24, 36, 37]. Thus, we should pay more attention on the prevention and control of children and adolescents’ hypertension.

This study also found the rural standardized prevalence of hypertension was higher than that of urban population. This is consistent with previous studies [38–40]. And the rural prevalence increased from 17.58% (2017) to 19.16% (2019), with the average annual increase rate of 0.53%. Hypertension epidemic increased rapidly in rural children and adolescents. It may be caused by the living environment (for example, latitude) [41], heredity and lifestyle and so on. We should develop further investigation to confirm the influencing factors. The rural children and adolescents’ hypertension problem deserves more emphasis and rural children and adolescents should be as a key population to work with. This requires more targeted intervention strategies to address the problem in children and adolescents.

In prefectures, the crude prevalence of hypertension in Pu’er increased from 18.05% (2017) to 22.53% (2019). The average annual increase rate was 1.49 percentage points. That of Kunming was 7.04% (2017) to 9.75% (2019). The average annual increase rate was 0.90 percentage points. We should make Pu’er and Kunming as a key area to carry out comprehensive health education intervention of children and adolescents blood pressure.

### Limitations

The present study has some limitations. Firstly, hypertension was defined as SBP and/or DBP at least 95th percentile based on age-, sex-, and height- percentiles. Although weight-for-height is not a perfect measure of body fat and blood pressure. Weight-for-height is highly correlated with body fat at the higher values for weight-for-height [20]. As a result, this study results may underestimate the prevalence of hypertension in 9–17 years old students. However, this study explored relative changes in its prevalence, regardless of what measures were used, the trend was apparent. Secondly, this study used data from three cross-sectional surveys, and each survey was conducted on different people every year. It is possible that unintentional errors occurred when estimating the prevalence of hypertension. Lastly, this study did not consider latitude.

## Conclusions

In conclusion, the blood pressure study in adults was of great concern, but truly little in children. This study focused on children and adolescents. And this serial cross-sectional study has indicated a slightly decreasing trend in the prevalence of hypertension among 9–17 years old children and adolescents in Yunnan from 2017 to 2019, whereas the prevalence trended to increase from 17.58% (2017) to 19.16% (2019) in rural areas. In viewing that childhood and adolescents’ hypertension can develop into adult hypertension and increase the risk of cardiovascular disease, childhood and adolescents’ hypertension should be considered as a major public health issue. We should conduct further studies to explore related risk factors to make an integrated intervention to prevent and control hypertension among children and adolescents in Yunnan, especially in rural areas.

## Data Availability

The database of this study is not accessible publicly. We have received administrative permission to access and use these data in the Ethics approval and consent to participate section. The raw data sets of this study can be available by emailing the corresponding author on reasonable request.

## Abbreviations

BP: Blood Pressure
SBP: Systolic blood pressure
DBP: Diastolic blood pressure

## References

[1] World Hypertension Day 2019 https://www.who.int/news-room/events/world-hypertension-day-2019. Accessed 25 May 2020. [2019]

[2] Zhou B, Bentham J, Cesare M (2017). Worldwide trends in blood pressure from 1975 to 2015: a pooled analysis of 1479 population-based measurement studies with 19.1 million participants. Lancet.

[3] Xing L, Liu S, Tian Y (2019). Trends in status of hypertension in rural northeast China. J Hypertens.

[4] John G, Nawab Q, Stuart P (2019). Raised blood pressure and risk of dementia. Eur Heart J.

[5] Chen W, Gao R, Liu L (2014). China cardiovascular disease report 2013(summary). Chin Circ J.

[6] Dong B, Wang Z, Song Y, Wang HJ, Ma J (2015). Understanding trends in blood pressure and their associations with body mass index in Chinese children, from 1985 to 2010: a cross-section observational study. BMJ Open.

[7] Oh JH, Hong YM (2019). Blood pressure trajectories from childhood to adolescence in pediatric hypertension. Korean Circ J..

[8] Drukteinis JS, Roman MJ, Fabsitz RR (2007). Cardiac and systemic hemodynamic characteristics of hypertension and prehypertension in adolescents and young adults the strong heart study. Circulation.

[9] Lande MB, Adams H, Falkner B (2009). Parental assessments of internalizing and externalizing behavior and executive function in children with primary hypertension. J Pediatr.

[10] Hao G, Wang X, Treiber FA, Harshfield G, Kapuku G, Su S (2017). Blood pressure trajectories from childhood to young adulthood associated with cardiovascular risk: results from the 23-year longitudinal Georgia stress and heart study. Hypertension.

[11] Howe LD, Chaturvedi N, Lawlor DA, Ferreira DL, Fraser A, Davey SG (2014). Rapid increases in infant adiposity and overweight/obesity in childhood are associated with higher central and brachial blood pressure in early adulthood. J Hypertens.

[12] Triosh A, Afek A, Rudich A, Percik R, Gordor B, Ayalon N (2010). Progression of normotensive adolescents to hypertensive adults: a study of 26,980 teenagers. Hypertension.

[13] Chen X, Wang Y (2008). Tracking of blood pressure from childhood to adulthood: a systematic review and meta-regression analysis. Circulation.

[14] Ingelfinger JR (2014). Clinical practice. The child or adolescent with elevated blood pressure. N Engl J Med.

[15] Su SY, Wang XL, Pollock JS (2015). Adverse childhood experiences and blood pressure trajectories from childhood to young adulthood: the Georgia stress and heart study. Circulation.

[16] Dong B, Ma J, Wang HJ (2013). The association of overweight and obesity with blood pressure among Chinese children and adolescents. Biomed Environ Sci.

[17] Poulter NR, Prabhakaran D, Caulfield M (2015). Hypertension. Lancet.

[18] CNHS. China health and nutrition survey: design and methods. See http://www.cpc.unc.edu/projects/china/about/design. Accessed 25 May 2020.

[19] Popkin BM, Du S, Zhai F, Zhang B (2010). Cohort profile: the China health and nutrition survey-monitoring and understanding socio-economic and health change in China, 1989-2011. Int J Epidemiol.

[20] National High Blood Pressure Education Program Working Group on High Blood Pressure in Children Adolescents (2004). The fourth report on the diagnosis, evaluation, and treatment of high blood pressure in children and adolescents. Pediatrics.

[21] Liang YJ, Xi B, Hu YH, Wang C, Liu JT, Yan YK (2011). Trends in blood pressure and hypertension among Chinese children and adolescents: China health and nutrition surveys 1991-2004. Blood Press.

[22] Lurbe E, Agabiti-Rosei E, Cruickshank JK, Dominiczak A, Erdine S, Hirth A (2016). 2016 European Society of Hypertension Guidelines for the management of high blood pressure in children and adolescents. J Hypertens.

[23] Flynn JT, Kaelber DC, Baker-Smith CM, Blowey D, Carroll AE, Daniels SR (2017). Clinical practice guideline for screening and management of high blood pressure in children and adolescents. Pediatrics..

[24] Dong YH, Ma J, Song Y (2017). National blood pressure reference for Chinese Han children and adolescents aged 7 to 17 years. J Hypertens.

[25] Gorostidi M, Vinyoles E, Banegas JR, de la Sierra A (2015). Prevalence of white-coat and masked hypertension in national and international registries. Hypertens Res.

[26] Kollias A, Ntineri A, Stergiou GS (2014). Is white-coat hypertension a harbinger of increased risk?. Hypertens Res.

[27] Ma SJ, Yang L, Zhao M, Xi B (2020). Changing trends in the levels of blood pressure and prevalence of hypertension among Chinese children and adolescents from 1991 to 2015. Zhonghua Liu Xing Bing Xue Za Zhi.

[28] Yang Y, Chang L, Lu C, Wei X (2016). The epidemiological characteristics of blood pressure among ethnic children and adolescents in Yunnan province. Chin J School Health.

[29] Zhao J, Yang Y, Chang L, Liu H, Yang F (2019). The blood pressure levels and influence factors in primary and middle school of Yunnan. Chin Health Educ.

[30] National High Blood Pressure Education Program Working Group on High Blood Pressure in Children and Adolescents (2004). The fourth report on the diagnosis, evaluation, and treatment of high blood pressure in children and adolescents [J]. Pediatrics.

[31] Jaber L, Eisenstein B, Shohat M (2000). Blood pressure measurement in Israeli Arab children and adolescents [J]. Isr Med Assoc J.

[32] Heeyeon Cho, Jae Hyun Kim. Secular trends in hypertension and elevated blood pressure among Korean children and adolescents in the Korea National Health and Nutrition Examination Survey 2007–2015. J Clin Hypertens. 2020; 00:1–8. 10.1111/jch.13842 PMC8029840 32175671

[33] Kit BK, Kuklina E, Carroll MD, Ostchega Y, Freedman DS, Ogden CL (2015). Prevalence of and trends in dyslipidemia and blood pressure among US children and adolescents, 1999-2012. JAMA Pediatr.

[34] Quaresma FRP, da Silva Maciel E, dos Santos Figueiredo FW, Adami F (2019). Factors associated with blood pressure disorders in Afro-descendant children and adolescents. BMC Pediatr.

[35] Chelo D, Mah EM, Chiabi EN, Chiabi A, Ndombo POK (2019). Prevalence and factors associated with hypertension in primary school children, in the Centre region of Cameroon. Transl Pediatr..

[36] Ingelfinger JR (2004). Pediatric antecedents of adult cardiovascular disease-awareness and intervention [J]. N Engl J Med.

[37] Chen X, Wang Y (2008). Tracking of blood pressure from childhood to adulthood: a systematic review and met a-regression analysis. Circulation.

[38] Liang X, Xiao L, Luo Y, Jiapei X (2020). Prevalence and risk factors of childhood hypertension in urban-rural areas of China: a cross-sectioal study. Int J Hypertens.

[39] Ebrahimi H, Emamian M, Hashemi H, Fotouhi A (2018). Prevalence of prehypertension and hypertension and its risk factors in Iranian school children. J Hypertens.

[40] Dong B, Wang Z, Ma J (2016). Urban-rural disparity in blood pressure among Chinese children:1985–2010. Eur J Public Health.

[41] Yang Yunjuan, Chang Litao, Lv Hui, Chen Lu, Wei Xijing, Liu Hong, Huang Dafeng, Huang Xin, Liu chunyan. Analysis on epidemiological characteristics and influencing factors of overweight, obesity in children and adolescents of different minorities in China. Chin J School Health 2016;37(8):1147–1150.

